# Deformation mechanisms in a coal mine roadway in extremely swelling soft rock

**DOI:** 10.1186/s40064-016-2942-6

**Published:** 2016-08-09

**Authors:** Qinghai Li, Weiping Shi, Renshu Yang

**Affiliations:** 1 State Key Laboratory of Mining Disaster Prevention and Control Co-founded by Shandong Province and the Ministry of Science and Technology, Shandong University of Science and Technology, Qingdao, 266590 China; 2 School of Mechanics and Civil Engineering, China University of Mining and Technology (Beijing), Beijing, 100083 China; 3 College of Geomatics, Shandong University of Science and Technology, Qingdao, 266590 China

**Keywords:** Deformation mechanism, Extremely swelling soft rock, Swelling potential, Field monitoring, Mechanical model, Plastic zone

## Abstract

The problem of roadway support in swelling soft rock was one of the challenging problems during mining. For most geological conditions, combinations of two or more supporting approaches could meet the requirements of most roadways; however, in extremely swelling soft rock, combined approaches even could not control large deformations. The purpose of this work was to probe the roadway deformation mechanisms in extremely swelling soft rock. Based on the main return air-way in a coal mine, deformation monitoring and geomechanical analysis were conducted, as well as plastic zone mechanical model was analysed. Results indicated that this soft rock was potentially very swelling. When the ground stress acted alone, the support strength needed in situ was not too large and combined supporting approaches could meet this requirement; however, when this potential released, the roadway would undergo permanent deformation. When the loose zone reached 3 m within surrounding rock, remote stress *p*_∞_ and supporting stress *P* presented a linear relationship. Namely, the greater the swelling stress, the more difficult it would be in roadway supporting. So in this extremely swelling soft rock, a better way to control roadway deformation was to control the releasing of surrounding rock’s swelling potential.

## Background

Roadway control in soft rock is a problem in many mines (Bilir 2011; Ghiasi et al. 2012; Serafeimidis and Anagnostou 2013; Thomas et al. 2013). For roadway control, a variety of support materials and structures have been developed, such as arch sheds, bolting, cables, and shotcrete (Goetze 1984; Okubo et al. 1984; Rotkegel 2001; Stalega 1995). As is necessary in situ, based on shed and bolting practice, new support structures have been developed, such as bolts with constant resistance under large deformation (He et al. 2013; He and Guo 2014; Sun et al. 2014), high-prestress and high-strength support systems (Kang et al. 2013; Wu et al. 2015), high-strength cable support systems (Li et al. 2012), round (Draganow et al. 1977; Gao et al. 2010) or square (Li et al. 2015) pipe supports filled with concrete, and grouted bolt systems (Srivastava and Singh 2015; Wang et al. 2009). For most geological conditions, combinations of two or more types could meet the requirements of most roadways. However, in some complex geological conditions, combinations of shedding, bolting, cabling, and shotcreting cannot control the deformation of surrounding rock.

The problem of roadway support in swelling soft rock has become a challenging problem in recent years (Bilir 2011; Ghiasi et al. 2012; Schädlich et al. 2013; Serafeimidis and Anagnostou 2013; Thomas et al. 2013). In swelling soft rock, support measures include either, the application of a strong, rigid supporting formwork to limit deformation, or allowing floor heave to release swelling pressures, or a combination of both (Christoph et al. 2011). In T13 tunnel, Ankara-Istanbul High-Speed Train Project, a heavier, non-deformable support system (NDSS) was applied in swelling and squeezing rocks (Aksoy et al. 2012). Another tunnel in Ankara-Istanbul High Speed Train Project, Tunnel 35, which was driven in a fairly weak and jointed rock, was also controlled by developed NDSS (Aksoy et al. 2014). In Canada, a tunnel situated in the Queenston Formation, South Ontario, was supported by a double-shell lining system, which included an initial lining of shotcrete, steel ribs, rock dowels, and a final lining consisting of a waterproof membrane and cast-in-place concrete (Ansgar and Thomas 2010). In a tertiary soft rock roadway in Liuhai coal mine, China, bolt-mesh-cable and double-layer-truss supports were used to control the large rheological deformation (Yang et al. 2015). Shen (2014) proposed a support system, which included an optimal cable/bolt arrangement, full length grouting, and high-load pre-tensioning of bolts and cables.

The No. 1 Mine in Chagannuoer (NMC), located in Xilin Gol League, Inner Mongolia, China, with a production capacity of 8.0 Mt/a, was under construction. Roof and floor of the main coal seam comprised extremely swelling soft rock. During excavation, the roadway deformed significantly and continuously. After being repaired repeatedly, it still could not be used normally. This problem increased infrastructure investment and delayed mine construction. To probe the mechanism of roadway deformation, based on the main return air-way in NMC, deformation monitoring and geomechanical analysis were conducted on site. At the same time, a plastic zone mechanical model, verified by physical experiments, was established. Based on the mechanical model, the necessary support strength was analysed, which provided guidance for such roadway support in future.

## Project profile

**Fig. 1:**
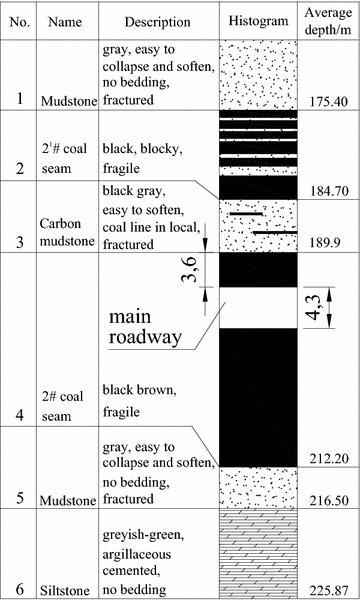
Histogram of coal seam and the location of the main return air-way

**Fig. 2:**
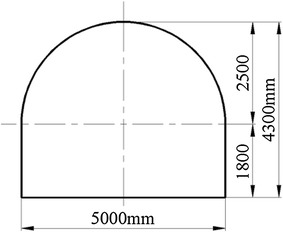
The main return air-way cross-section

**Fig. 3:**
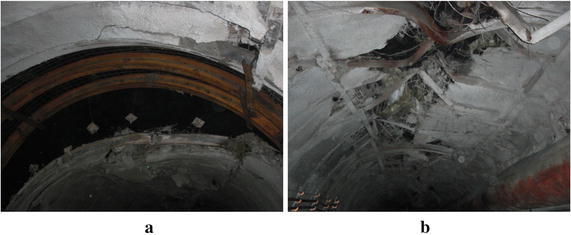
In situ damaged status. **a** Roof subsidence. **b** Top arch distortion

In Chagannuoer coal field, there were five fully or partially minable coal seams, with thicknesses ranging from 1.60 to 59.60 m (28.92 m on average). In this coal field, two mines were designed and NMC, with its production capacity of 8.0 Mt/a, was the first mine under construction. In NMC, the No. 2 coal seam, at 22.3 m thickness on average, belonged to lignite and lay between Cretaceous and Jurassic strata, was the main mining seam and was buried at a depth of 212.2 m. Roof and floor of the No. 2 coal seam were primarily mudstone and carbon mudstone with extremely low strengths (Table 1), which were lower than the strength of coal seam and were in a loose, fractured state. In mine design, considering lower strength of roof and floor, the main roadway was placed in No. 2 coal seam (Fig. 1). Using the return-air roadway as example, it was designed as a straight wall-semicircular arch type (Fig. 2). The height of its straight wall was 1.8 m and the diameter of the semicircular arch was 5.0 m. The net cross-sectional area of the roadway was 18.82 m^2^. During the return air-way excavation, the mining pressure was exceeding large and floor heave, two sides shifting closer and roof subsidence presented almost all the time. Damaged forms of roadway are shown in Fig. 3. Several supporting approaches (Table 2) have been tested on site, but almost all failed and the roadway needs to be repaired all the time to maintain normal use, which brings risk to mine safety and production.

**Table 1:** Mechanical parameters of the strata

Name	Bulk density (average)/(t/m^3^)	Porosity (%)	Compressive strength (average)/MPa	Tensile strength (average)/MPa	Apparent cohesion (average)/MPa	Internal friction angle/(°)
Mudstone/carbon mudstone	1.66–2.14 (2.0)	26.20–54.11	0.12–5.12 (1.71)	0.06–0.39 (0.12)	0.24–0.39 (0.31)	25.16–29.01
Siltstone	2.01–2.65 (2.2)	17.48–27.13	0.64–8.00 (2.26)	0.07–0.61 (0.34)	0.23–0.50 (0.38)	13.70–25.50
Coal	1.16–1.35 (1.3)	25.40–45.72	1.18–9.44 (3.44)	0.35–1.13 (0.56)	0.42–1.02 (0.66)	27.05–32.03

**Table 2:** Support approaches used on site

Order	Support approaches	Yielding support?
1	36 U-shape sheds	Yes
2	36 U-shape sheds + anchoring and shotcreting	Yes
3	Pair of 16 I-shape sheds + anchoring and shotcreting	No
4	Pair of 12 mine-used I-shape sheds + anchoring and shotcreting	No

## Geomechanical analysis

### Field monitoring

**Fig. 4:**
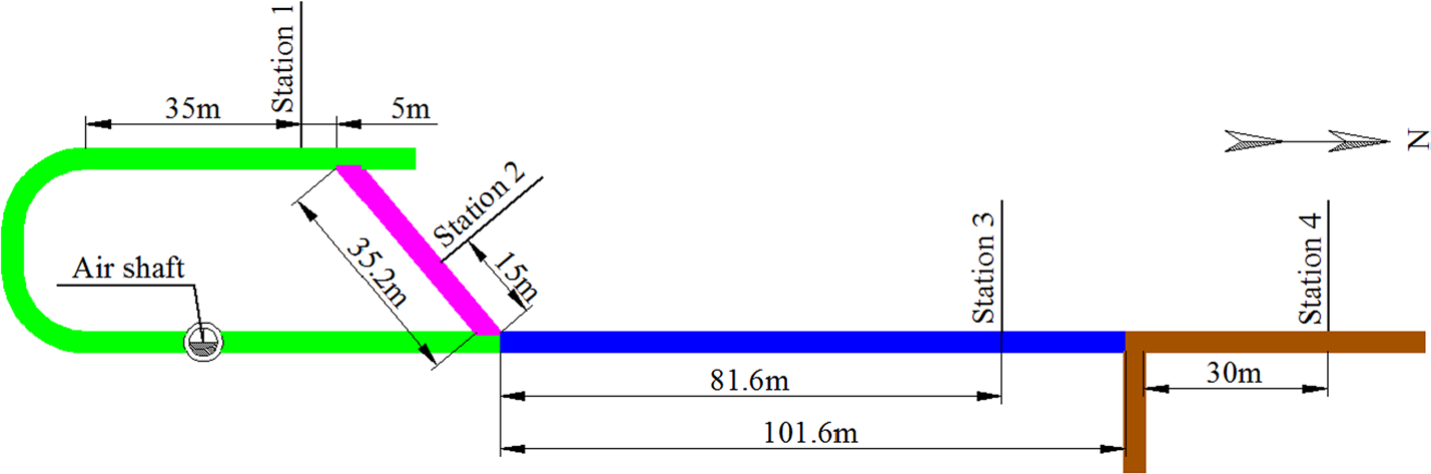
Monitoring stations in the return air-way

**Fig. 5:**
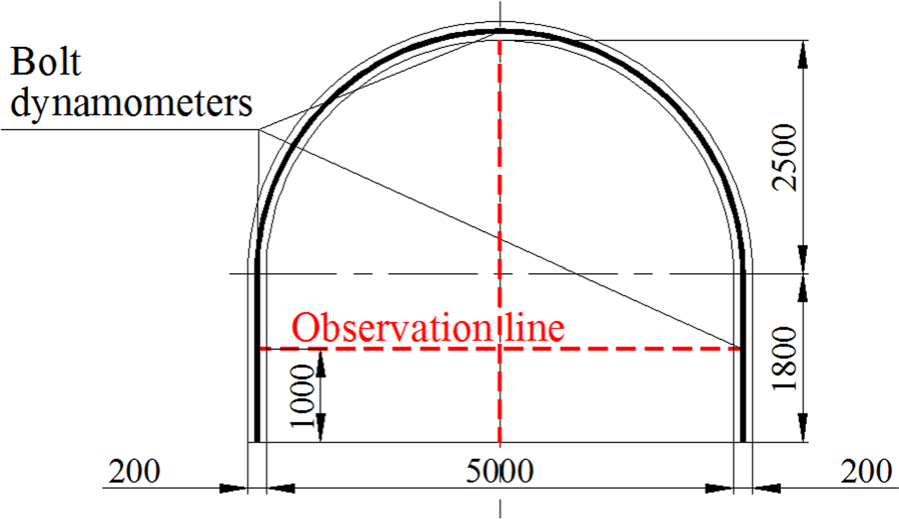
Layout of observation lines (unit: mm)

To understand the controlling effect of different supporting approaches listed in Table 2, four monitoring stations were established in the return air-way (Fig. 4). The monitoring stations were numbered 1–4. Roadways in different colours were supported by different approaches. From station 1–4, the roadway was supported by method of 1–4 (listed in Table 2) correspondingly. At each station, displacements of roof to floor and left to right side were monitored (Fig. 5).

**Fig. 6:**
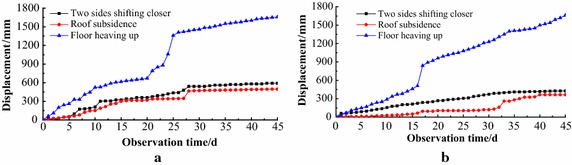
Displacements at station 3 and 4. **a** Station 3. **b** Station 4

Large deformation occurred in four stations. Data at stations 3 and 4, which benefitted from greater support strength than stations 1 and 2, were analysed (Fig. 6). At stations 3 and 4, the maximum rate of roof subsidence reached 20 and 15.1 mm/d. By the end of monitoring, roof subsidence reached 496 and 366 mm in two stations respectively. Roof shotcrete cracked and fell off, and the top arch was flattened and distorted. The maximum rate of two sides moving reached 19.3 and 12.5 mm/d. By the end of monitoring, two sides squeezing inward reached 596 and 426 mm. Over time, the deformation rate decreased gradually, but the deforming never stopped.

Based on the design of releasing pressure, there was no support on floor. Under this status, floor heaving in stations 3 and 4 were almost the same. The maximum rate of floor heaving reached 38.3 and 31.9 mm/d at these two stations. During monitoring, the floor heave even reached 1658 and 1660 mm in two stations.

From monitoring results, it was found that, in this soft coal seam, roadway deformed significantly and the deforming never stopped.

### Geological conditions and ground stress

Within the scope of mining area, all coal seams were stable, with a very gentle dip angle. There was a slight syncline (20 km in length, 4–10 km in width) in mining area. In general, the geological conditions in this area were simple.

To find the magnitude of ground stress, measurements were conducted at three locations in situ. From the results it was found that the maximum principal horizontal stress (*σ*_*h*max_) was 8.41–8.66 MPa, with 12.5–18.1° to horizontal plane. The minimum principal horizontal stress (*σ*_*h*min_) was 2.54–3.25 MPa, with −13.34–6.58° to horizontal plane. The vertical stress (*σ*_*v*_) was 4.72–4.91 MPa. The ratio between *σ*_*h*max_ and *σ*_*v*_ were approximately 1.8.

### Compositions

Laboratory tests showed that the mudstone in both roof and floor was mainly composed of quartz, potassium feldspar, plagioclase, and clay minerals. Among the measured compositions, the clay mineral reached 60.6 % in content (see Table 3). Worse still was that the clay minerals were mainly highly swelling montmorillonites, illites, and kaolinite. In these clay minerals, the montmorillonite accounted for 82.0 %. Compositions of clay minerals are shown in Table 4. Correspondingly, in both roof and floor mudstones, the montmorillonite reached 49.7 % in content. According to the swelling soft rock classification criteria proposed by Sun et al. (2005), roof and floor mudstones in NMC were classified as extremely swelling soft rock.

**Table 3:** Mineral compositions of mudstones

Mineral compositions	Quartz	Potassium feldspar	Plagioclase	Clay minerals
Property (%)	22.7	11	5.7	60.6

**Table 4:** Mineral compositions of clay fraction

Clay mineral compositions	Montmorillonite	Illite	Kaolinite
Property (%)	82	10	8

### Swelling pressure

**Fig. 7:**
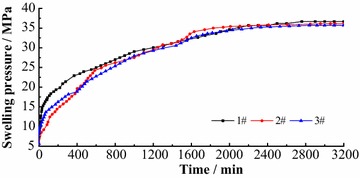
Swelling pressures versus time

To quantify the swelling pressure arising in this soft rock, three specimen swelling tests were conducted in laboratory. Each specimen was cylindrical, of 50 mm diameter and 50 mm high. During testing, three blocks were placed in experimental apparatus and immersed in water. Curves of swelling pressure are shown in Fig. 7.

When absorbing water, the pressure increased rapidly, and then increased exponentially, but the swelling rate decreased gradually. Up to 10 h after immersion, the three blocks exhibited certain differences in swelling. Thereafter, the three blocks swelled almost the same to each other. Blocks reached complete saturation after 52 h immersion, by when the swelling pressure was 35.7–36.7 MPa.

Contrasting all engineering geological conditions in situ, it was found that the geological structure in this coal field was simple, and the ground stress was not abnormal. But the mudstone exhibited large swelling pressure in laboratory tests. Due to rigid constraints and complete saturation in tests, the swelling pressure was large, and much larger than in situ ground stress. Almost no constraints and lack of water in situ, the swelling pressure will be less than the measured values in laboratory tests. However, from test results it was found that there was significant swelling potential in this extremely swelling soft rock.

## Mechanical analysis on plastic zone in extremely swelling soft rock

### Mechanical model

**Fig. 8:**
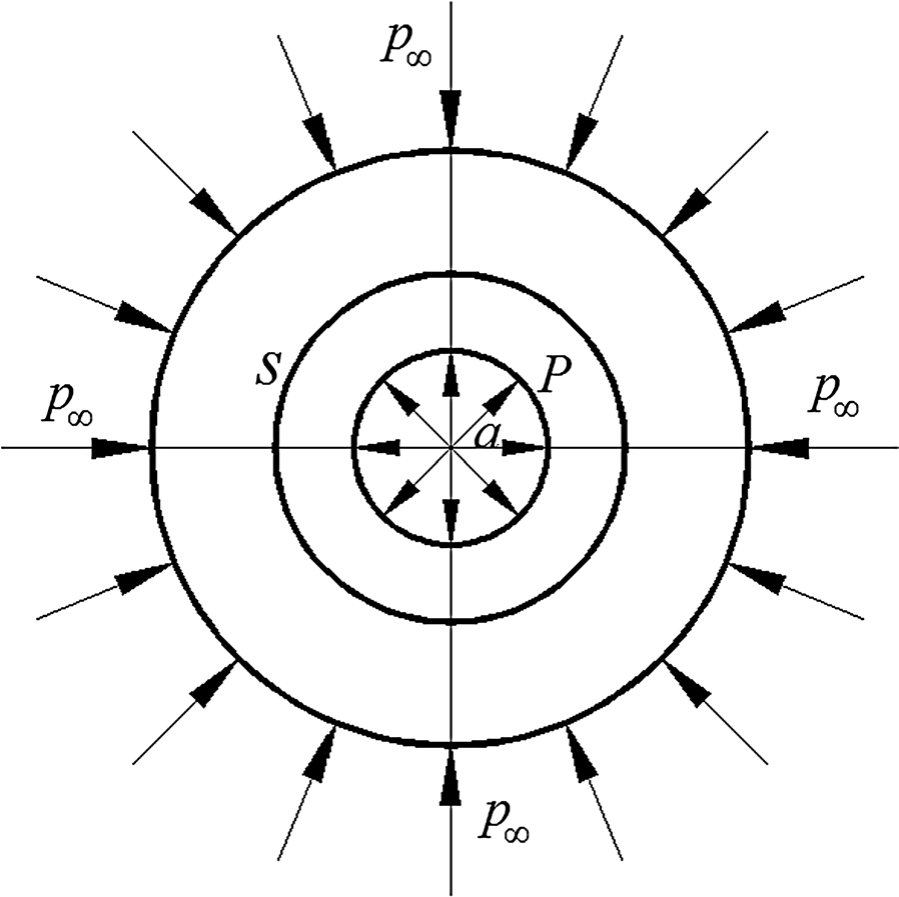
Mechanical model of roadway

Roadway in type of straight wall-semicircular arch is complicated in mechanical analysis. To simplify analysis, the roadway is designed as a circle (*r* = *a*) in mechanical model. Strata at infinity bear an isotropic pressure *p*_∞_. A positive pressure *P*, simulating the support strength, is applied on the roadway surface (Fig. 8). The mechanical model is axisymmetric and the displacement is purely radial. There are only in-plane stress components *τ*_*rr*_(*r*) and *τ*_*θθ*_(*r*).

Due to loose and fractured characteristics of extremely swelling soft rock, the strata demonstrated particulate characteristics. Referred to mechanics (Howell et al. 2009), yield criterion of granular rock is

1 $$ 2(\tau_{rr} \tau_{\theta \theta } )^{1/2} \le - \cos \varphi (\tau_{rr} + \tau_{\theta \theta } ) $$

in which, *φ* is internal friction angle of rock.

Formula (1) can be changed as

2 $$ k\tau_{\text{rr}} = \tau_{\theta \theta } $$

in which, $$ k = \frac{1 + \sin \phi }{1 - \sin \phi }. $$

In plane polar, Navier equation (Howell et al. 2009) is

3 $$ \frac{{d\tau_{rr} }}{dr} + \frac{{\tau_{rr} - \tau_{\theta \theta } }}{r} = 0 $$

On roadway surface and in the far field, the boundary conditions satisfyWhen *r* = *a*, *τ*_*rr*_ = −*P*When *r* → ∞, *τ*_*rr*_ → −*p*_*∞*_, *τ*_*θθ*_ → −*p*_*∞*_While the rock keeps elastic, the constitutive relations (Howell et al. 2009) are

$$ \tau_{rr} = \left( {\lambda + 2\mu } \right)\frac{{du_{r} }}{dr} + \lambda \frac{{u_{r} }}{r}, $$

4 $$ \tau_{\theta \theta } = \lambda \frac{{du_{r} }}{dr} + \left( {\lambda + 2\mu } \right)\frac{{u_{r} }}{r} $$

in which, *λ* is Lame constant, *μ* is shear modulus and *u*_r_ is radial displacement.

Based on boundary conditions, combined with (3) and (4), the stress components are gained as

$$ \tau_{rr} = - p_{\infty } + \left( {p_{\infty } - P} \right)\frac{{a^{2} }}{{r^{2} }}, $$

5 $$ \tau_{\theta \theta } = - p_{\infty } - \left( {p_{\infty } - P} \right)\frac{{a^{2} }}{{r^{2} }} $$

On roadway surface (*r* = *a*), based on (2) and (5), the yield will occur when

6 $$ P = \frac{{2p_{\infty } }}{1 + k} $$

Plastic zone boundary is set as *r* = *s*. The elastic solution in *r* > *s* can be gained as *P* replaced by 2*p*_∞_/(1 + *k*) and *a* replaced by *s* in (5). Namely

$$ \tau_{rr} = - p_{\infty } + p_{\infty } \left( {\frac{k - 1}{{k{ + }1}}} \right)\frac{{s^{2} }}{{r^{2} }}, $$

7 $$ \tau_{\theta \theta } = - p_{\infty } - p_{\infty } \left( {\frac{k - 1}{k + 1}} \right)\frac{{s^{2} }}{{r^{2} }} $$

Meanwhile, in plastic region *r* < *s*, based on boundary conditions, combined with (2) and (3), the stress is gained as

8 $${\tau _{rr}} =  - P{\left( {\frac{r}{a}} \right)^{k - 1}}$$

When *r* = *s*, combined with *k*, (7) and (8), the loose zone *s* can be decided as

9 $$ s = a\left(\frac{{(1 - \sin \varphi )p_{\infty } }}{P}\right)^{{\frac{1 - \sin \varphi }{2\sin \varphi }}} $$

The plastic zone, gained by (9), started from centre of roadway. In supporting design, the plastic zone was always regarded as starting from the surface of roadway, so the actual plastic zone *s’* should be (*s* *−* *a*). Namely

10 $$  s' = a\left(\frac{{(1 - \sin \varphi )p_{\infty } }}{P}\right)^{{\frac{1 - \sin \varphi }{2\sin \varphi }}} - a $$

### Rationality analysis on mechanical model

To verify the rationality of above mechanical model for plastic zone analysis in extremely swelling soft rock, based on main return air-way in NMC (supported by closed 36U-shape sheds was chosen to be analysed), a physical experiment was conducted and roadway deformation process was reappeared.

## Experiment design

### (1) Model size

In laboratory, the frame size length, width, and height were 1600, 400, and 1600 mm. According to main return air-way in NMC, the roadway was 5000 mm in width and 4300 mm in height (Fig. 2). Considering influenced zone by mining, the geometry ratio *C*_*l*_ between prototype and experiment model was set to 16. Because of shed thickness and exposed anchors on roadway surface, the roadway size was enlarged by 5 mm in both width and height in experiment. Namely the roadway in experiment was 318 mm wide and 274 mm high.

### (2) Strata materials

Carbon mudstone strata were formed by gypsum and water. Coal seam was formed by gypsum, water and additive. Rock layers were paved layer-by-layer to simulate bedded deposition in situ. According to similarity theorems (Yuan 1998), the volume weight ratio *C*_*γ*_ and stress ratio *C*_*σ*_ between prototype and experiment model were defined as:

11 $$ C_{\gamma } = \frac{{\gamma_{p} }}{{\gamma_{m} }} $$

12 $$ C_{\sigma } = C_{\gamma } \, \times \,C_{l} $$

in which, *γ*_*p*_ and *γ*_*m*_ are volume weight of strata in situ and in experiment model respectively.

The volume weight of physical strata and actual rock was more close to each other, so *C*_*γ*_ was defined as 1.176. Correspondingly, *C*_*σ*_ was 18.82 (*C*_*l*_ = 16). The physical strata, in which the bulk densities were in the ratio *C*_*γ*_ to actual rock, and the compressive strengths, tensile strengths and apparent cohesions were in the ratio *C*_*σ*_ to actual rock, were found after repeated testing. The internal friction angle *φ* of physical coal seam, tested in laboratory, was nearly 27°.

### (3) Loading mode

According to ground stress and *C*_*σ*_ 18.82, *σ*_*h*max_ was almost 0.46 MPa and *σ*_*v*_ was almost 0.26 MPa in experiment. During loading, horizontal and vertical stresses were increased simultaneously. The vertical stress was increased in 0.1 MPa increments every 30 min and the horizontal stress was applied as the vertical stress multiplied by 1.8 (*σ*_*h*max_/*σ*_*v*_ was 1.8). When the vertical stress reached 0.30 MPa, and the horizontal stress was 0.54 MPa, there were no damaged signs in surrounding rock. It can be determined that, when ground stress acted alone, the roadway, supported by approaches listed in Table 2, could not deform as much. So the ground stress was not the cause of roadway deformation in this extremely soft rock.

And then, based on existing loads, the vertical and horizontal stresses increased simultaneously in 0.1 MPa increments every 30 min. The model was not damaged until the loads reached 0.8 MPa in vertical and 1.04 MPa in horizontal.

### (4) Shed material

**Fig. 9:**
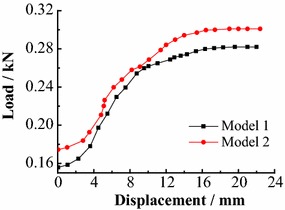
Test data of model shed bearing capacities

Steel bar, 2 mm in thickness and 10 mm in width, was chosen for the material of sheds in experiment. Two model sheds were tested and their capacities were shown in Fig. 9. During testing, the loading mode was the same as that determined previously. The capacity of the model shed was 0.291 kN (average of two tests). In situ, sheds were installed every 700 mm along roadway. According to geometry ratio *C*_*l*_, the sheds in experiment were placed every 44 mm along roadway. Combined with 115 mm height of two sides, the support strength offered by these sheds on two sides was 0.0575 MPa in physical model.

## Experimental results

**Fig. 10:**
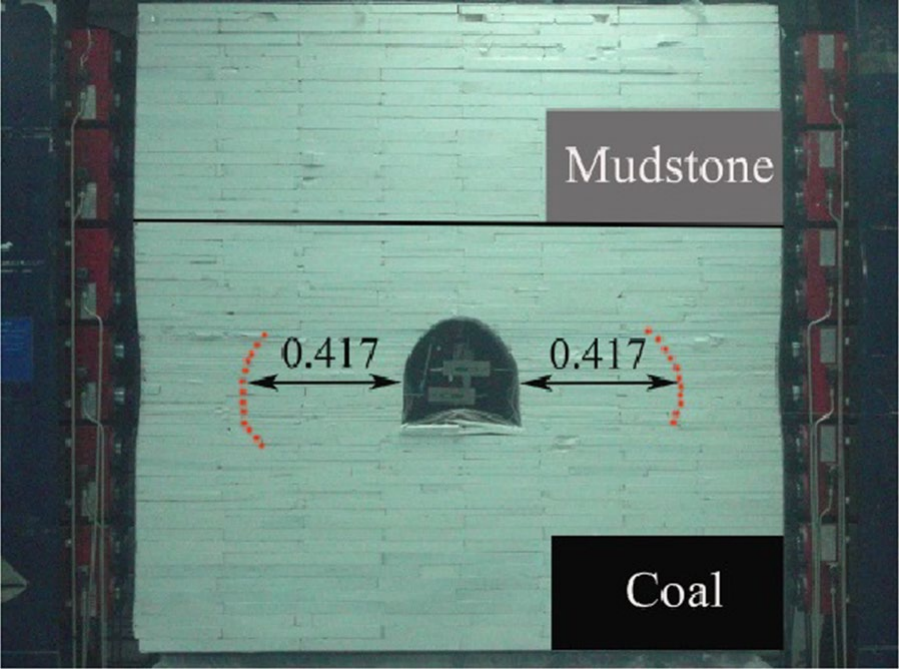
Damage mode in the model

When vertical stress reached 0.7 MPa and horizontal stress reached 0.94 MPa in experiment, the loose zone in two sides extended to 0.417 m (Fig. 10). With *φ* = 27°, *p*_∞_ = 0.94 MPa and *a* = 0.159 m, according to formula (10), the plastic zone was calculated as 0.434 m. Compared with experimental result, the error of calculated one was 4.1 %. So the mechanical model was deemed suitable for plastic zone analysis in extremely swelling soft rock.

### Support strength analysis

**Fig. 11:**
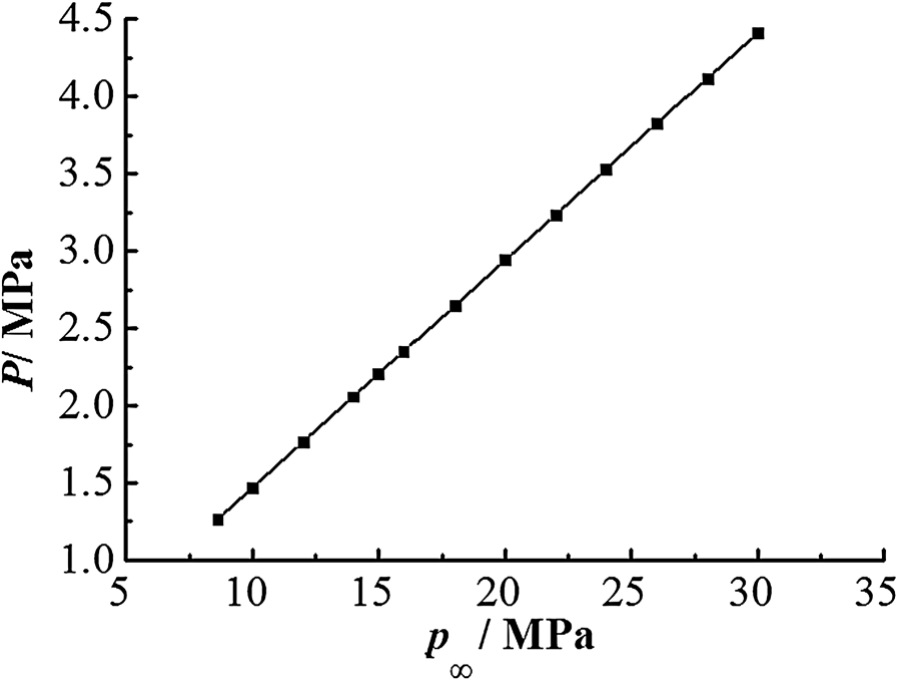
Relationship between *p*_∞_ and *P*

In extremely swelling soft rock, according to formula (10), when the loose zone controlled much smaller, the required support strength will be much larger. While *a* is 2.5 m and *φ* is 27°, as well as the loose zone reaches 3 m in surrounding rock, the relationship between *p*_∞_ and *P* (Fig. 11) is given by:

13 $$ P = \, 0. 1 4 7p_{\infty } - \,{ 2}. 3 2 5 { } \times { 1}0^{ - 4} $$

From formula (13), as *p*_∞_ increases, the needed support strength *P* grows linearly. When *p*_∞_ is 8.6, 10, 15 and 20 MPa, the support strength *P* will be 1.265, 1.471, 2.207 and 2.943 MPa correspondingly. So when the ground stress acts alone, the support strength needed in maintaining roadway stability is 1.265 MPa, which is not too large and the support approaches listed in Table 2 could meet the requirement. However, when the remote stress *p*_*∞*_ increases to 15 MPa (mainly induced by surrounding rock swelling), the support strength will exceed 2 MPa, which would need extremely high-strength support structures to meet this requirement. Furthermore, the greater the swelling stress, the more difficult will be in roadway support. So the better way to control roadway deformation is to control the releasing of swelling potential. In this extremely swelling soft rock, when supports yielded and swelling stress released, more conditions would be created for rock swelling. So the non-deformable support system (NDSS) would be an effective way for roadway control in this extremely swelling soft rock.

## Conclusions

To understanding the roadway deformation mechanisms in extremely swelling soft rock in NMC, monitoring and geomechanical analysis were conducted on site, and a plastic zone mechanical model was established. The following conclusions were drawn: the soft rock had significant potential to swell. When ground stresses acted alone, the support strength needed in situ was not too large and combined support approaches could meet this requirement. When swelling potential of this rock released, the roadway would deform significantly and the deformation would be permanent. Based on mechanical analysis, when the loose zone reached 3 m in surrounding rock, remote stress *p*_*∞*_ and supporting strength *P* presented a linear relationship. Namely, the greater the swelling stress, the more difficult would be in roadway support. So in this extremely swelling soft rock, a better way to control roadway deformation was to control the releasing of surrounding rock’s swelling potential. And the non-deformable support system (NDSS) was an effective way for roadway control in this extremely swelling soft rock.
